# Dynamic Personalized Optimization: An AI Functionality Framework for Digital Therapeutics

**DOI:** 10.2196/75256

**Published:** 2026-03-18

**Authors:** Dohyoung Rim

**Affiliations:** 1 Rowan Corporation, 18F Yonsei Severance Bld, 10, Tongil-ro, Jung-gu, Seoul, 04527, Republic of Korea, 82 2-6731-0810, 82 2-6930-5606

**Keywords:** dynamic personalized optimization, digital therapeutics, AI functionality framework, personalized treatment, large language models

## Abstract

Dynamic Personalized Optimization (DPO) is introduced as a conceptual framework that defines core artificial intelligence (AI) functions required to deliver real-time, personalized, and optimized treatment in digital therapeutics (DTx). DPO continuously refines therapeutic strategies by integrating patient data, treatment content, usage feedback, and status measurements to provide real-time, personalized treatment. Using predictive AI models, DPO adapts treatment approaches based on patient responses, thereby improving therapeutic effectiveness. Furthermore, this paper explores the potential role of large language models (LLMs) in supporting DPO by processing diverse and complex data formats. By addressing current limitations in real-time personalization within DTx, DPO establishes a structured, AI-driven approach to delivering tailored digital interventions. This framework ultimately aims to enhance treatment efficacy and improve patient engagement.

## Introduction

Digital Therapeutics (DTx) are software-based interventions designed to prevent, manage, or treat medical conditions and provide direct therapeutic benefits through digital technology [1]. Such digital interventions are particularly important for neurological disorders, which require complex, personalized approaches [2]. Neurological disorders such as tinnitus, depression, and mild cognitive impairment require individualized care that DTx can offer [3,4], but the full potential of DTx is realized only with artificial intelligence (AI) integration [5].

These DTx solutions can continuously monitor patients, deliver real-time feedback, and dynamically adjust treatments to improve outcomes [6]. AI plays a crucial role in DTx by analyzing large-scale patient data to enable real-time personalization and improved treatment outcomes [3,7,8].

This study introduces a functional framework for AI in DTx, which supports real-time personalization through dynamic adaptation. Existing DTx lack a formal framework for real-time personalization. Prior work has identified persistent challenges in dynamic personalization [8,9], including the need for more adaptable models and limitations of static content in DTx. This paper addresses this gap by introducing a new framework called Dynamic Personalized Optimization (DPO).

## DPO Framework

### Definition and Characteristics of DPO

DPO is a conceptual framework that delineates the core AI functions necessary for DTx to achieve dynamic personalization and optimal therapy. It integrates and analyzes patient data to refine treatment strategies in real time. DPO tailors treatment plans by incorporating patient data and responses to offer individualized therapeutic approaches. A predictive AI model anticipates the patient’s future status and optimizes treatment accordingly. DPO dynamically optimizes personalized therapy by integrating clinical data, feedback, and treatment content.

Building on just-in-time adaptive interventions [10], DPO formalizes relevant data types and introduces adaptive treatment content optimization through AI-based prediction of patient feedback or status. While traditional just-in-time adaptive interventions primarily focus on determining the timing of interventions, DPO differentiates itself by continuously optimizing treatment content through AI-based prediction of patient feedback, thereby extending the concept from temporal adaptation to content-level personalization.

### Necessity of DPO in DTx

Neurological and other complex disorders require personalized, responsive treatment. DPO is essential to achieving this in DTx, as it continuously tailors therapy based on patient-specific data in real time.

### The Four Data Types of DPO

**Figure 1.**
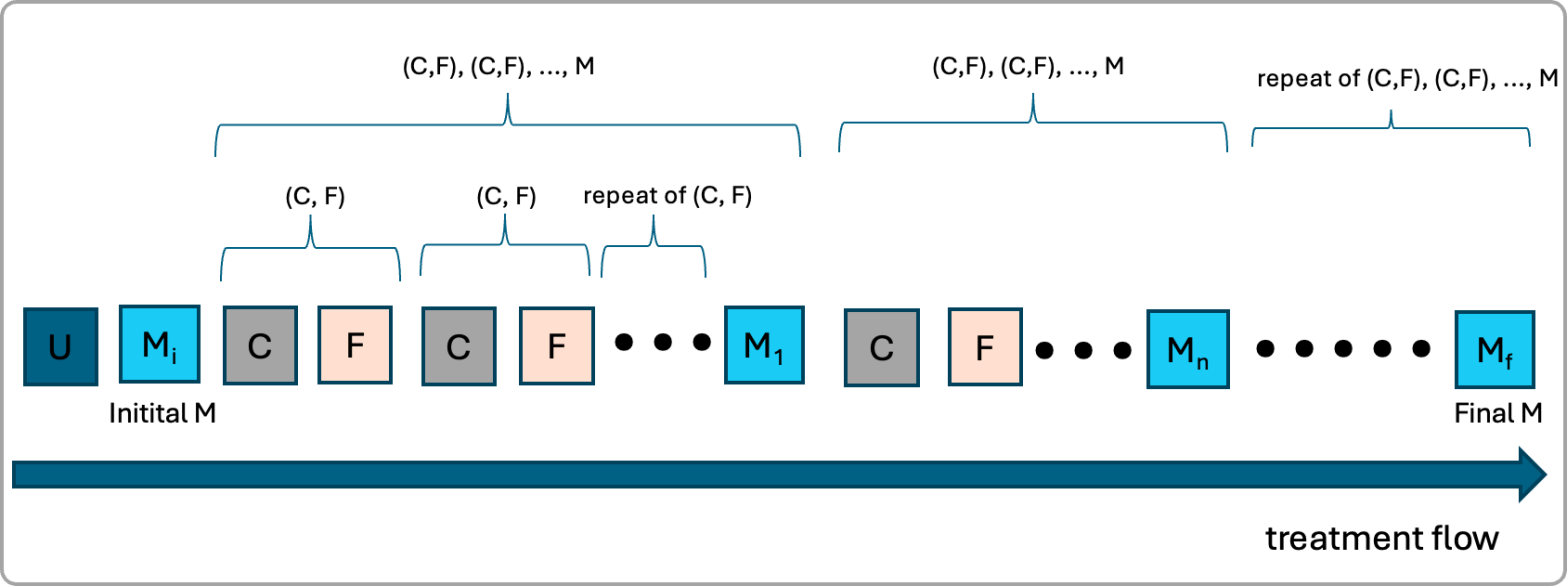
The treatment process begins with a sequence of 4 data types: initial user data (U), initial patient status (Mi), and a repeated cycle of treatment content (C) and feedback (F), which leads to updated status measurements (M). This treatment-feedback sequence and patient status update continues throughout the treatment period.

DPO uses four types of data to tailor therapy. User data (U) include information about the patient, such as demographics, medical history, and lifestyle. Status measurement (M) data refer to objective health metrics, including symptom severity and test results. Treatment content data (C) encompass information related to the therapy itself, such as descriptions, types, and formats. Finally, feedback data (F) consist of feedback received after therapy, such as treatment game scores or the patient’s reported feelings. AI in DTx uses these 4 types of data to create personalized treatment plans. Figure 1 illustrates how these 4 data types are sequentially processed throughout the therapeutic process.

## Adaptive Treatment Content Optimization

Within the DPO framework, AI is expected to continuously select the treatment content (C) that yields the greatest improvement in a patient’s final status (M_f_) relative to the initial status (M_i_). However, identifying the “best” content in advance for AI model training is nearly impossible because it varies by individual and context and cannot be known until tried.

### Predicting F

**Figure 2.**
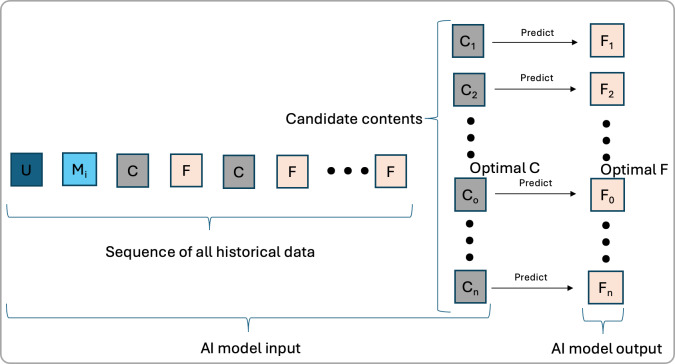
One optimization approach involves predicting the expected feedback (F) for each candidate content. The optimal content (C_o_) is selected based on the highest predicted F. An artificial intelligence (AI) model is trained on historical data sequences to predict F for a given candidate C.

As Figure 2 illustrates, instead of directly predicting the optimal content C, this approach focuses on predicting F for a given C. In a DTx environment, real-world data collection yields sequential data (U, M, C, and F) throughout the treatment. An AI model can be trained on these historical data sequences to predict subsequent F. Although F does not directly represent treatment efficacy, it informs future treatment decisions. This approach assumes that feedback (F) is a valid proxy for treatment efficacy. Prior research shows a strong correlation between user engagement in digital interventions and improved treatment outcomes [11], though engagement alone may not fully capture clinical efficacy. Nonetheless, leveraging this correlation, the model can predict F for candidate treatment contents and select the optimal one (C_o_) with the highest predicted F.

### Predicting M

**Figure 3.**
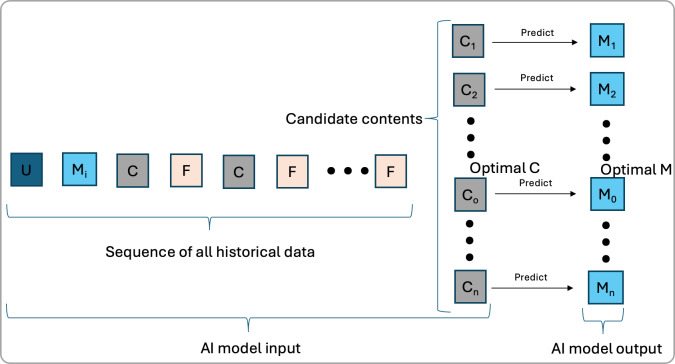
An alternative approach involves predicting the final patient status (M_f_) for each candidate content. The artificial intelligence (AI) model takes historical data and candidate C as input and predicts the resulting status (M_f_). The optimal content (C_o_) is selected to maximize improvement in M_f_ relative to the initial status.

Figure 3 illustrates another approach: predicting the patient’s M_f_. In this approach, an AI model is trained with prior data sequences as input and M_f_ as the output. If the model learns this relationship accurately, it can predict M_f_ for a given input sequence and a candidate C. By predicting M for all candidate C options, the model can select the C_o_ that most effectively improves M.

Together, these approaches eliminate the need to explicitly label C as the correct answer. Instead, AI predicts F or M_f_ to dynamically determine the optimal C, ensuring that patients engage with the most effective therapeutic content. However, implementing these AI-driven strategies in practice requires managing complex and heterogeneous DTx data, which poses significant challenges.

In a cognitive intervention context for mild cognitive impairment (MCI) [12], DPO operates as follows. The system first collects U such as demographic and behavioral information including age, education level, daily activity, and sleep patterns. It also gathers initial measurement data (M_i_), such as the Mini-Mental State Examination score [13] representing the patient’s baseline cognitive state. Based on these inputs and the history of previously delivered C—for example, cognitive games that train memory, attention, or problem-solving skills—and corresponding F, such as game scores reflecting engagement and task performance, the AI model predicts either the expected F or the postsession M for each candidate content option. The predicted value then guides the system in selecting the next C_o_ for the patient’s subsequent training session. This process of providing C and receiving F repeats over time, allowing the system to refine personalized cognitive training. Through this iterative mechanism, DPO continuously optimizes therapeutic content to enhance cognitive function and engagement in patients with MCI.

## Challenges of Data in DTx and LLM Strategy

### Overview

DTx data appear in diverse forms—text (eg, clinical notes), numeric measurements, images, and video—varying widely in length and quality. This heterogeneity hinders uniform processing of data by AI. Addressing this issue requires careful data preprocessing, stringent quality control, and AI models capable of integrating disparate data types.

LLMs have significant potential to overcome data processing challenges in DTx [14]. The following strategies describe how LLMs can support DPO.

### Integrated Processing of Various Data Formats

LLMs can process diverse data formats (including text, numerical data, and images) thereby enabling comprehensive analysis to improve treatment outcomes [15]. They analyze unstructured text, such as medical records and patient feedback, to extract key details and interpret numerical data like vital signs and laboratory results. Additionally, LLMs use embedding techniques to represent each data modality as a numeric vector, enabling integrated analysis across different data types. This approach allows processing of disparate data types within the same mathematical space.

### Processing Variable Data Sizes

LLMs can handle data inputs of widely varying lengths [16,17]—from brief notes and detailed medical records to extensive patient histories and multisource health logs. They can also process time-series data to identify patterns and trends in symptom logs and vital signs. This capability enables real-time monitoring and more adaptive care.

## Discussion

The DPO framework proposed in this study has been designed as a structural model applicable to a range of DTx scenarios, offering a systematic functional definition of AI capabilities required for the personalization and adaptive coordination of digital therapeutic content. In DPO, LLMs are not used to generate new therapeutic content but rather serve as analytical tools to integrate and interpret multimodal data for personalized optimization. This paper presents a conceptual design framework and does not include empirical validation for 2 primary reasons. First, DPO is formulated as a high-level functional abstraction rather than a model-specific architecture, and its applicability may be realized through various algorithmic implementations. Therefore, the focus of this work is on the completeness and coherence of the framework design rather than the comparative performance of specific models. Second, the DPO framework is currently undergoing pilot-level integration in several ongoing DTx development pipelines within an industry setting, and early experimental outcomes related to these efforts are planned to be reported in a separate study. Future work will empirically evaluate the implementation and performance of each functional component of the DPO framework—prediction, personalization, and optimization—within actual digital therapeutic contexts using real user data. Additional studies will also explore the generalizability and scalability of DPO across diverse clinical domains, including MCI, depression, and tinnitus, to further establish the framework’s practical utility and robustness.

## Conclusion

The proposed DPO framework offers a structured approach to operationalizing AI in DTx, with the potential to improve both engagement and therapeutic efficacy. Integrating LLMs into DPO could greatly enhance personalized care delivery and improve health care outcomes. At the same time, ensuring data privacy, security, and safety in digital therapeutics remains essential. Moreover, the responsible use of AI-based DTx requires addressing ethical issues such as transparency, fairness, cybersecurity, and data protection, which must be implemented with accountability [18]. Recent studies on responsible AI deployment further emphasize the importance of governance, transparency, and privacy as key foundations for ethical implementation in health care [19,20]. AI must generate transparent recommendations while ensuring clinical validation and ethical oversight. Overcoming these challenges could enable a new level of personalized treatment and substantially improve patient outcomes. Leveraging advanced AI models for DPO may facilitate the integration of heterogeneous patient data in ways previously unattainable, essentially serving as a “brain” for DTx that learns and adapts to each patient. Going forward, the practical implementation and clinical validation of DPO (potentially with LLMs) are crucial next steps. These efforts must be accompanied by careful attention to ethical and regulatory considerations. If DPO can maintain patient engagement by tailoring content to what is therapeutically optimal for each individual, the therapeutic efficacy of DTx could be substantially enhanced. Such personalized adaptation prevents patient dropout or disengagement caused by monotonous or ineffective content [1], thereby further improving outcomes.

## Abbreviations

AI: artificial intelligence
C: treatment content data
C_o_: optimal content
DPO: Dynamic Personalized Optimization
DTx: digital therapeutics
F: feedback data
LLM: large language model
M: status measurement data
MCI: mild cognitive impairment
M_f_: final status
M_i_: initial status
U: user data
